# Exploring health workers’ perspectives on factors affecting patient experience in emergency caesarean section response time: a qualitative study in hospitals in Makassar City, Indonesia

**DOI:** 10.1186/s12913-025-13294-4

**Published:** 2025-10-03

**Authors:** Ayu Rizky Ameliyah, Stang Stang, Irwandy Irwandy, Fridawaty Rivai, Muhammad Arsyad

**Affiliations:** 1 Doctoral program of Public Health Sciences, Faculty of Public Health, Hasanuddin University, Makassar, Indonesia; 2 Department of Hospital Administration, Faculty Health Technology, Megarezky University, Makassar, Indonesia; 3 Present Address: Department of Statistics, Faculty of Public Health, Hasanuddin University, Makassar, Indonesia; 4 Department of Hospital Management, Faculty of Public Health, Hasanuddin University, Makassar, Indonesia; 5 Department of Health Promotion and Behavioral Sciences, Faculty of Public Health, Hasanuddin University, Makassar, Indonesia

**Keywords:** Patient experience, Emergency caesarean, Response time, Indonesia

## Abstract

**Background:**

The achievement of an emergency caesarean response time of ≤ 30 min in Indonesia is still far below the target. This can impact the patient experience. This study aims to explore the factors causing delays in emergency caesarean response time and quality improvement strategies to address this issue.

**Method:**

This qualitative study was conducted in five hospitals in the city of Makassar, Indonesia, from November 2024 to January 2025. The phenomenological approach, along with in-depth interviews, was employed in this research. Data were collected through semi-structured interviews. A total of 14 healthcare workers were interviewed using purposive sampling, consisting of obstetricians, general practitioners, midwives, and nurses, and the data were analyzed using thematic analysis. The inclusion criteria for selecting informants are healthcare professionals who possess knowledge and expertise in the field of emergency cesarean sections, as well as having at least two years of work experience handling patients with such cases in hospitals.

**Results:**

This study identifies four main factors that can impact the patient experience in achieving emergency caesarean response time, namely: system factors, patient and family factors, staff factors, and internal hospital policy factors. This study also produces quality improvement strategies to achieve emergency caesarean response times across various health professions, such as the importance of patient and family education, emotional support, the need for comprehensive hospital facilities to ensure the smooth operation of emergency caesarean procedures, the need for periodic performance evaluations of healthcare workers regarding response time achievements and most importantly the availability of documented, socialized, and actively used standard operating procedures by all healthcare workers.

**Conclusion:**

This study provides a comprehensive understanding of the factors that cause delays in achieving emergency cesarean response times. This can have both positive and negative impacts on the patient experience. Therefore, policy support and commitment from various health professions are essential for the successful implementation of achieving emergency cesarean response times in Indonesia.

**Supplementary Information:**

The online version contains supplementary material available at 10.1186/s12913-025-13294-4.

## Introduction

A new paradigm within the International Hospital Accreditation Standards, that healthcare services focus on patient-centered care. The implementation of patient-centered care applications can be understood through patient experience regarding what patients perceive and the extent to which their perceptions, satisfaction, and needs are addressed during their treatment in the hospital [1–3]. Patient experience is a series of all care service processes received in the form of practice, managerial, and clinical care received by patients [4]. By examining various aspects of patient experience, one can assess the extent to which patients receive care that respects and is responsive to the individual patient, their needs, and values [5]. One of the treatments that requires a positive patient experience is emergency caesarean patients, who are more vulnerable to experiencing stress [6–9]. A good experience while at a healthcare facility can encourage further use of the healthcare facility, while a bad experience can reduce the willingness to use the healthcare facility again [10]. Maternal death is the second leading cause of death after the HIV virus among women aged 15–49 [11]. In Indonesia, the maternal mortality rate currently stands at 140 per 100.000 live births [12], which is twice the target set by the Sustainable Development Goals (SDG), namely less than 70 per 100.000 live births by 2030. This condition indicates that Indonesia still faces significant challenges in reducing the national maternal mortality rate. Preventing maternal mortality, especially during childbirth, has become a concern both globally and nationally [13, 14]. More than 62% of maternal and infant deaths occur in hospitals during the labor and postpartum phases [15, 16]. Often, response time becomes the main contributor that can save mothers and newborns [17].

Based on the 2022 Performance Accountability Report of the Ministry of Health of the Republic of Indonesia, only 58 out of 331 hospitals (17.52%) managed to meet the target response time of ≤ 30 min for emergency cesarean procedures, indicating a still low achievement of the quality indicators for maternal emergency services. Meanwhile, at the local level, specifically in the city of Makassar, out of all the hospitals listed in the Ministry of Health’s report, only one hospital managed to meet that standard in 2022 [18]. However, that one hospital is not part of our research location. The hospital that was the research location actually showed various obstacles that resulted in delays in response time. These findings were obtained from interviews with healthcare workers and reflect conditions consistent with national trends.

This research is one of the first known studies in Indonesia to adopt factors that influence patient experience related to response time for emergency cesarean sections and to discuss quality improvement strategies to address this issue. On the other hand, various previous studies have focused on measuring the response time of patients undergoing emergency cesarean sections in relation to maternal outcomes [19–21], yet the researchers still reference earlier works to strengthen this study. Because, to the best of our knowledge none has examined the factors causing delays in response time that could impact the experience of emergency caesarean patients from the perspective of various professions in the hospital, to bridge this gap the researchers employed qualitative research methods to gather information related to the causative factors and quality improvement program. This study aims to explore the factors causing delays in emergency caesarean response time and quality improvement strategies to address this issue. The importance of this research can be used as a basis to improve the quality of healthcare services in emergency obstetric situations, create a better patient experience, and enhance the safety of mothers and babies.

## Methods

This research uses a qualitative method with a phenomenological approach and in-depth interview technique. The phenomenological approach was chosen to understand the meaning of informants’ experiences related to response time delays in handling emergency cesarean sections. The reason for using this approach is that the phenomenological approach focuses on understanding experiences as directly felt by the individuals who experience them. This method assumes that humans create meaning from every experience, and their narratives reflect that meaning in depth [22]. Additionally, in-depth interviews are used in this research, the reason for choosing this method is to explore information in detail and depth, as well as to minimize the influence of other informants that usually occurs in group discussions. It can be said that if our focus is on the experiences directly encountered by the participants and the phenomenological approach will be our methodological framework, then with this type of research, in-depth interviews are very appropriate to use because they allow participants to narrate their experiences in detail and depth [23]. The central phenomenon addressed in this study is the delay in response time in handling emergency cesarean sections in hospitals, which can impact patient experiences from the perspectives of various healthcare professionals.

### Study design and participants

This study uses a qualitative research design with a purposive sampling technique. The inclusion criteria for selecting informants are that the participants selected as key informants are those who possess knowledge and expertise in the field of emergency caesarean sections. Additionally, the informants are healthcare workers who have at least 2 years of experience handling emergency caesarean patients in the hospital. The exclusion criteria include informants who express unwillingness, such as refusing informed consent after being explained to over the phone, as well as healthcare workers who have not been directly involved in emergency caesarean cases in the past six months. In this context, the selection of participants is based on recommendations from the hospital’s human resource management department where the research is conducted. Furthermore, to support the government’s programme in improving the quality of healthcare services, this research is conducted in five hospitals, consisting of four government-owned hospitals and one private-owned mother and child hospital as a comparison. Among the four government hospitals, two are general hospitals, and the other two are mother and child hospitals. Based on the classification of hospital types, three of them are classified as type B and two as type C.

Overall, 18 participants were selected, including those who work as obstetricians, general practitioners in the emergency department, midwives, and nurses. They were contacted by phone to convey the purpose and benefits of the research and to request their willingness to be interviewed virtually (online) or face-to-face. In the end, 14 participants from five hospitals in Makassar City, Indonesia confirmed their willingness to participate in this research and there are 4 people who refused to participate. The maximum variation sampling technique was considered in the selection of participants [24], where participants were chosen from various professional backgrounds, ages, work experiences, and types of hospitals to capture diverse perspectives on the emergency cesarean process. Participant information is provided in (Supplementary File 2). The number of informants in each hospital ranged from two to three people from various professions. Data saturation was reached at the 13th interview, where the information obtained began to repeat and no longer produced significant new themes. To strengthen the validity of the findings, the researcher conducted one additional interview, bringing the total number of participants to 14. This step was taken to ensure that no important perspectives were missed and to affirm the consistency of the data. This is supported by the research of Guest et al. (2020), which states that conducting several additional interviews after the saturation point is a recommended practice to confirm that no new themes emerge [25].

### Interview methods

**Fig. 1:**
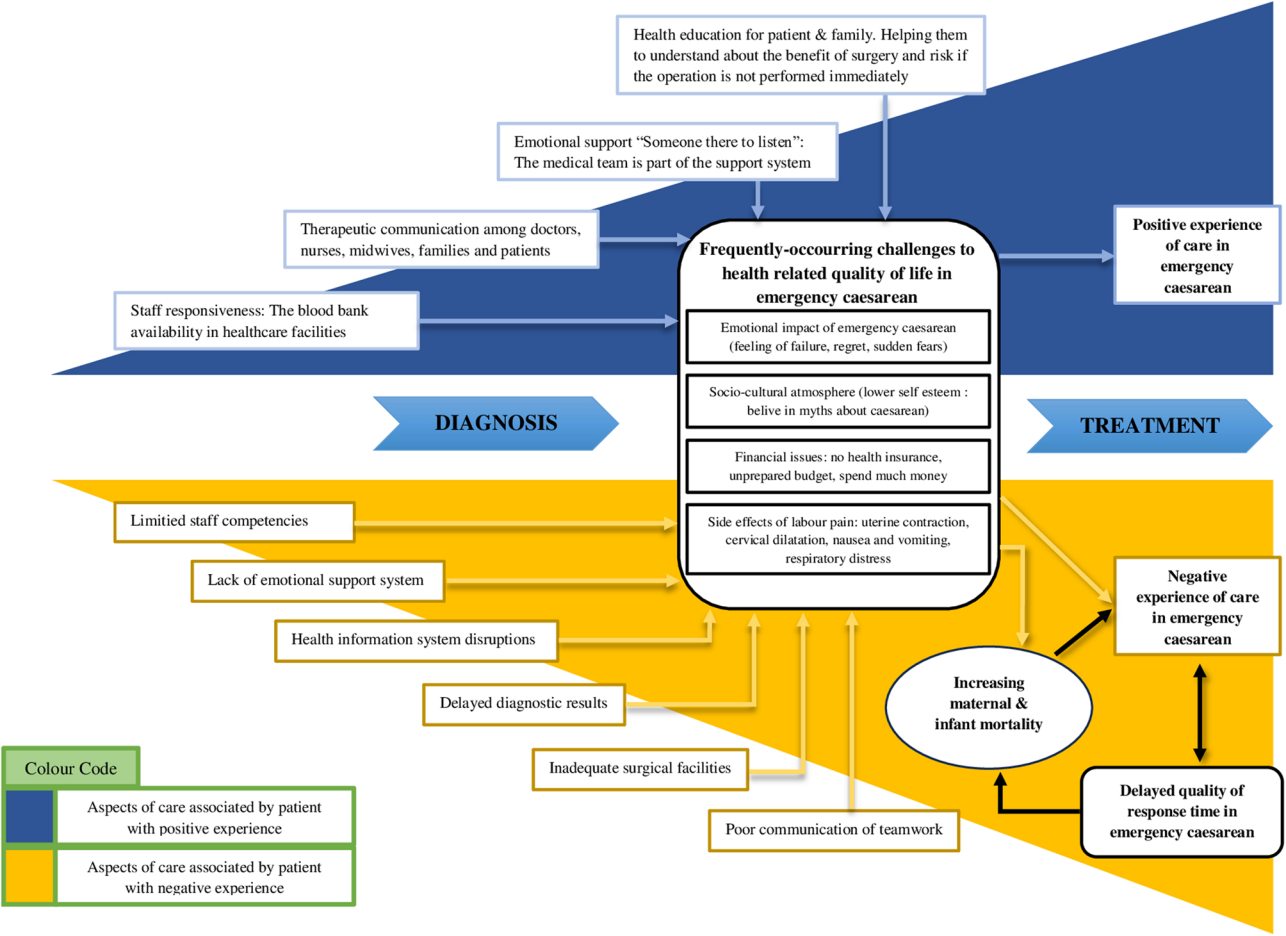
Model of factors affecting quality of patient experience in emergency caesarean

This qualitative research was conducted in Makassar City, Indonesia between November 2024 and January 2025. This research was conducted using semi-structured interviews that involved discussions on key questions regarding the factors causing delays in the response time for emergency caesarean patients. The closing stage ended with a summary of the discussion, and the researcher provided an opportunity for informants to offer recommendations for improving services, so that patients can have a good experience during their treatment. The interviews was developed by adopting the sonis theory (2019) factors that influence patient experience in the emergency department [26]. The interview summary is illustrated by adopting the concept of Wagland et al. (2016) on factors influencing patient experience [27], but the concept of this research focuses on factors affecting quality of patient experience in emergency caesarean as shown in (Fig. 1).

The interview guide used in this study is based on the interview questions provided as an additional file (Supplemental Material 1). Besides being based on theory, this interview guide was developed by the researcher and finalised after consulting with the supervising lecturers on campus, who are also lecturers in the field of hospital management and have researched various studies related to patient experiences. The interviews, recorded in audio, were conducted in Indonesian and lasted between 30 and 60 min. Prior consent was obtained from the participants before recording the interview process, and neither the participants nor the researchers had any prior relationship. In order to ensure the validity of the data and enhance the validity of the findings, the researcher held two meetings with the participants. The member checking process was conducted after the preparation of the interview transcripts [28]. The transcript was shown again to each participant for confirmation and verification. Participants were given the opportunity to make corrections or add information to ensure that the recorded data truly represented their experiences. To ensure the security and confidentiality of the data, all interview recordings are stored in an encrypted folder on the researcher’s personal computer, which is password-protected, with access limited to the principal researcher. Audio recordings will be stored for a maximum of two years after publication as a precaution in case clarification is needed in the future. After the period has passed, all recording data will be permanently deleted to protect the privacy of the participants and adhere to the principles of qualitative research ethics. All procedures in this study were approved by an ethical committee from the Ethics Committee of the Faculty of Public Health, Hasanuddin University with an approval number 2881/UN4.14.1/TP.01.02/2024.

### Statistical analysis

Data were analyzed using a thematic analysis approach according to Braun and Clarke’s (2006) [29]. The researcher identified main themes and subthemes, as well as explored recurring themes throughout the interviews to help explain the research findings in an easily understandable manner. After that, the analysis results from the lead researcher are compared and discussed collaboratively by all authors of this journal to reach a final agreement. This process is carried out to ensure consistency of meaning, enhance the validity of findings, and minimize individual bias in data interpretation. For participant codes, they are assigned alphabetically based on their profession.

All recorded data is transcribed verbatim manually and entered into an interview table for the data cleaning process. The interview transcripts are coded based on predetermined categories. The coding process is carried out by the researcher using an interview matrix in Microsoft Excel. The interview table consists of information such as participant characteristics, participant responses and data reduction, as well as summaries of each theme. The results of the categories were re-examined by two research assistants using an interview matrix to ensure the validity of the data interpretation. The presentation of qualitative data uses direct quotes from respondents, narratives, and images.

## Results

### General characteristics of participants

This study consists of 14 women (100%) as participants, in Indonesia, women have a greater interest in reproductive health and childbirth, making it very rare for this profession to be undertaken by men. For hospital affiliation, 8 participants (57.1%) came from women and child hospitals, while 6 participants (42.9%) came from general hospitals. The work experience of obstetricians ranges from 6 to 16 years, indicating a high level of experience, while general practitioners range from 2.6 to 3 years. Midwives have an even higher level of experience, ranging from 5.6 to 27 years, and nurses also have significant experience, ranging from 14.6 to 15.4 years (Table 1, Supplemental Material 2). Based on the results of interviews with 14 participants there are five themes emerged from this interview, including: (1) factors caused by the system, (2) factors caused by patients and families, (3) factors caused by staff, (4) factors caused by hospital policies, and (5) recommendations to address response time delays to improve positive patient experiences during emergency caesarean sections. A total of 22 sub-themes were identified for each factor (Table 2).

**Table 1:** Characteristics of participants

Code	*n*	Profession	Age (y)	Work Experience in Hospital (y)
Obgyn A	1	Obstetrician-Gynecologist	38	6.0
Obgyn B	2	Obstetrician-Gynecologist	51	10.0
Obgyn C	3	Obstetrician-Gynecologist	61	16.0
Obgyn D	4	Obstetrician-Gynecologist	39	6.5
Doctor A	5	General Practitioner	30	2.6
Doctor B	6	General Practitioner	30	3.0
Midwife A	7	Midwife	34	9.3
Midwife B	8	Midwife	48	27.0
Midwife C	9	Midwife	47	17.0
Midwife D	10	Midwife	57	13.0
Midwife E	11	Midwife	47	5.6
Midwife F	12	Midwife	39	16.0
Nurse A	13	Nurse	44	14.6
Nurse B	14	Nurse	36	15.4

**Table 2:** Themes extracted from the interviews

Main Theme	Sub-Theme
	Exploring Factors Affecting Patient Experience In Emergency Caesarean Section Response Time
System Factors	The limited availability of operating rooms Slow laboratory results The unavailability of a blood bank in the hospital Information system network constraints
Patients & Family Factors	The patient is determined to have a normal delivery. Patient Anxiety Delayed family consent (informed consent) The patient is not accompanied by family Financial Issues Delivery equipment that is not yet ready
Staff Factors	Slow Time Management Poor teamwork communication Staff’s lack of skills
Factors of Internal Hospital Policy	The lack of availability of Standard Operating Procedures
Recommendation to address response time delays	Emotional Support Patient and Family Education Family communication via telephone Therapeutic Communication Staff-Patient Blood supply that is always available at the hospital Responsive IT staf Emergency patient priority level Special box for emergency caesarean

### System of factors

The majority of informants highlighted that various systemic barriers in healthcare facilities also affect the delay in response to emergency cesarean actions. These barriers reflect the weak support of infrastructure and work facilities in hospitals, which directly impact the speed of decision-making and the implementation of emergency medical actions. Here are interviews with several informants:

#### Operating room limitations

Constraints such as limited operating rooms, especially when the patient load is full or facilities are inadequate in remote areas, often cause delays in emergency surgeries and endanger patients’ lives.

*“The most common issue is obstacles such as full operating rooms*,* especially when the number of attending physicians is quite high. When all the operating rooms are in use and there is an urgent need (cito)*,* the surgery schedule often has to be postponed.” (Obgyn A)*.

*The lack of availability of operating rooms remains a problem in several areas*,* especially in remote or island regions. For example*,* during my visit to West Java*,* a case of delayed emergency treatment led to adverse obstetric outcomes. This was caused by the limited operating rooms*,* where a bone fracture surgery was taking place at that time. Although the patient had been treated for preeclampsia*,* the condition worsened because the operating room was not ready.” (Midwife B)*.

#### Slow network infrastructure

Additionally, the network frequently encounters problems. Several times there have been issues when the network does not function properly, causing the data that has been input not to be sent successfully.

*“The problem that often occurs is the network. Several times*,* issues arose because the network was not functioning properly. When we had already typed the data*,* the information had not been sent.” (Doctor A)*.

#### Laboratory examination

In laboratory tests before surgery, there are seven basic tests that must be conducted, one of which is a routine blood test that takes about 30 min. Meanwhile, tests such as triple elimination and blood chemistry generally take longer.

*“There are seven mandatory basic tests*,* such as routine blood tests that take about 30 minutes*,* while triple elimination and blood chemistry tests usually take longer.”* (Nurse B).

#### The unavailability of a blood bank

Blood management through the Indonesian Red Cross is crucial, especially if the patient’s HB level is below 10 because doctors will not proceed with the procedure without the availability of blood. Considering there is no blood Bank on-site, the fastest time to obtain blood is usually around 1 h, but it can take 2 to 3 h depending on the queue at the Indonesian Red Cross.

“*Blood management through the Indonesian Red Cross is very important*,* especially if the patient’s HB is below 10*,* because the doctor will not proceed without ready blood*,* especially since there is no blood Bank here. The fastest time is usually 1 hour*,* but it can take 2–3 hours depending on the queue at the Indonesian Red Cross.”* (Midwife C).

### Patient and family factors

Internal factors affecting patients and families also contribute to delays in response time for emergency cesarean actions. These factors include a strong desire for normal childbirth influenced by cultural and mythological beliefs, delayed approval resulting from sudden decisions, insufficient financial readiness, and a lack of family support during the decision-making process. Here are interviews with several informants:

#### Determined to give birth naturally

Some patients are still influenced by myths and refuse a cesarean section, even in emergency conditions like a breech baby that should be handled immediately, they insist on giving birth normally.

*“There are patients who are still influenced by myths and refuse a cesarean section*,* even in conditions like a breech baby that requires emergency action*,* They still insist on wanting to give birth normally”.* (Midwife A)

#### Patient anxiety

The most common issue that arises is when patients receive the decision for surgery, because many of them feel shocked and become stressed when facing the sudden situation.

*“The problem is*,* when the patient has to undergo surgery*,* the patient feels shocked and stressed.”* (Doctor B).

#### Delayed family approval (informed consent)

The process of obtaining consent for medical procedures is often hindered, partly because the patient’s husband is not present when decisions need to be made and is late in signing the informed consent. Additionally, the patient’s limited understanding of the surgical procedure makes them tend to wait for approval from their husband or parents first.

*“First*,* the patient’s husband was not present when the decision was made. Second*,* when the patient’s husband finally arrived*,* he was late to sign the informed consent.”* (Obgyn D).

*“We often face cultural barriers. Most of the patients who come here have a lower-middle social*,* economic*,* or educational background*,* so their understanding of the section procedure is still limited. As a result*,* they often wait for approval from their husband or parents first.”* (Obgyn B).

#### Unprepared finances

Because they do not have insurance yet, some patients feel anxious about the costs that need to be incurred, making financial conditions one of the reasons they hesitate to undergo treatment.

*“There are still many patients without insurance*,* which means economic issues sometimes make patients here afraid of high payments.”* (Nurse A).

#### Patient not accompanied by family

In some cases, the patient is not accompanied by family, so there is no authorised party to sign the informed consent for medical procedures.

*“There are no family members accompanying*,* they cannot sign the informed consent themselves. However*,* often the one accompanying is not a family member for example a boyfriend. In such cases*,* they cannot sign documents on behalf of the patient.”* (Midwife D).

#### Delivery equipment that is not yet ready

Another obstacle is the case of delayed childbirth occurring because the patient arrives without bringing essential items such as a change of clothes, baby nappies, or other personal equipment. As a result, the childbirth process must be postponed until the family brings these items to the hospital.

*“Delays in the delivery process often occur because the delivery equipment has not been prepared when arriving at the hospital. So*,* they have to wait for family members to bring the necessary items*.” (Doctor A).

### Staff factors

The interview results indicate that staff-related obstacles, such as delays, ineffective communication, and low skill levels, contribute to poor patient experiences and potentially hinder the achievement of response times for emergency caesarean sections. Here are interviews with several informants:

#### Slow time management

The factor of medical personnel also poses a challenge, such as the delay of the anaesthesiologist or the absence of the responsible doctor. This causes a delay in medical procedures, whereas in emergency situations, the speed of handling is very important.

*“Staff factors also become obstacles*,* such as the anesthesiologist arriving late or the responsible doctor not being present”.* (Obgyn C)

#### Hierarchical communication between residents and supervising doctors

Teaching hospitals also often encounter several obstacles, for example: medical decision-making cannot be done directly. Patient condition information must go through several reporting stages. This tiered procedure sometimes causes the handling to become slower.

*“This hospital is an educational hospital*,* some conditions indeed require the reporting process to be done in stages*,* starting from junior residents to mid-level residents*,* then to chief residents*,* and finally reported to the responsible doctor.”* (Doctor A).

#### Lack of teamwork communication

Barriers in communication and teamwork often arise during shift changes, as information is not always effectively conveyed between staff members. This impacts the timeliness of responses to patient conditions, especially in situations requiring quick intervention.

*“Communication and teamwork often become obstacles*,* especially during shift changes. This causes slower response times.”* (Nurse A).

#### Staff skills deficiency

One of the causes of delays in medical actions is the lack of midwives’ competence, especially those who have never received training related to maternal and neonatal emergencies. This unpreparedness slows down decision-making when facing emergency cases.

*“Delays are often caused by a lack of skills*,* especially for midwives who have never attended maternal and neonatal emergency training*,* making it difficult to make decisions.”* (Midwife F).

### Internal hospital policy factors

One participant also highlighted the internal policy, namely the Standard Operating Procedures available in hospitals.

#### Lack of availability of standard operating procedures related to emergency caesarean

In the review of the Emergency Operational Procedures in four regencies/cities in Indonesia, it was found that three of the regions merely copied the document without a deep understanding.

*“I am currently reviewing standard operating procedures*,* and in the review of emergency standard operating procedures in 4 districts and cities*,* it was found that 3 areas have standard operating procedures that were merely copied without deep understanding. Even the doctor who presented it was confused because the standard operating procedures were created solely for accreditation purposes*,* not based on their actual needs or practices.”* (Midwife E).

### Strategy program for improving patient experience quality to achieve emergency caesarean response time

The efforts are needed to address delays in handling emergency caesarean patients. This strategy requires a holistic approach, starting from patient engagement such as emotional support and patient education to operational management. The most important aspect is that participants from various professions in five hospitals also provided recommendations for quality improvement strategies to create a good experience for emergency caesarean patients related to response time (Table 3 and 4).

**Table 3:** Summary of interview results by profession

	Theme	Obstetrician-Gynecologist	General Practitioner	Midwife	Nurse
*Exploring Factors Affecting Patient Experience In Emergency Caesarean Section Response Time*	System Factors	The limitation of operating rooms and the sufficient number of doctors	Network issues that often occur, especially during medication prescriptions.	Blood management at the Indonesian Red Cross takes up to 3 h because there is no blood bank at the hospital.	The laboratory test results are delayed due to a shortage of staff and a high number of patients.
	Patient & Family Factors	Cultural barriers of patients and low socioeconomic status lead to slow family consent.	The patient felt shocked and stressed before the surgery. Delivery equipment that is not yet ready.	There are patients who refuse a cesarean section because of myths. Minors must be accompanied by family to sign the informed consent.	The husband arrived late and signed the informed consent. There are still many uninsured patients who are worried about the cost of a cesarean section.
	Staff of Factor	The anesthesiologist or ob-gyn who arrived late	Hierarchical reporting from junior residents to the attending physician	Lack of skills, especially among midwives who have not undergone maternal and neonatal emergency training.	Ineffective team communication during shift changes in the emergency department
	Internal Hospital Policy Factors	N/A	N/A	Lack of Standard Operating Procedures for emergency caesarean	N/A

**Table 4:** Summary of interview results by profession

	Theme	Obstetrician-Gynecologist	General Practitioner	Midwife	Nurse
*Exploring Factors Affecting Patient Experience In Emergency Caesarean Section Response Time*	Recommendation to address response time delays	The need for patient education about the indications, risks, and benefits of emergency caesarean sections. Setting the most urgent priorities. If there are simultaneous emergencies, prioritize the most critical one.	Emotional support and reinforcement from doctors are important for the effectiveness of treatment. Doctors need to provide effective communication and involve the family. The availability of a special emergency caesarean box containing clothing, medicine, and medical equipment ensures quick and efficient handling.	Emotional support from the medical team to prevent postpartum blues or depression. Time efficiency through communication via WhatsApp or video call to family for approval	Therapeutic communication between nurses, doctors, families, and patients Blood bank available at the hospital The IT medical record staff is available 24 h to address network issues.

## Discussion

Our research highlights the participants’ views that the factors in achieving emergency caesarean response time are influential on patient experience, which can be either negative or positive. There are four factors that can cause issues with response time. The first includes system factors, this research reveals that the limited number of operating rooms poses a significant obstacle in the management of emergency cesarean sections. When the number of obstetric specialists increases without a corresponding addition of operating rooms, the risk of delayed procedures becomes higher, including in emergency situations. This condition disrupts the smooth flow of services and directly impacts the response time, which ideally should be quick to avoid complications for both the mother and the baby [16, 30]. Findings from other informants also reinforce the previous statement that the limited number of operating rooms is the main obstacle in handling emergency caesarean sections. The cases of delays that occurred in the western part of Indonesia during the informants’ visits to several hospitals indicate that the limitations of facilities, especially in remote areas, have a serious impact on the safety of mothers and babies [31]. The diversity of geographical conditions and the lack of infrastructure in various regions also contribute to significant disparities in the delivery of healthcare services between regions. These factors can cause delays in decision-making and preparation for cesarean operations, resulting in lower adherence to guidelines [32, 33].

In addition, technical issues such as network disruptions were also identified as obstacles to the smooth delivery of emergency caesarean services. When the information system does not function optimally, the data input and prescription processes become hindered, requiring alternative manual communication via telephone. This condition shows that full dependence on digital systems without backup mechanisms can disrupt service speed [34]. The laboratory examination process is also one of the factors that affect the smooth handling of emergency caesarean sections. The limited number of laboratory staff and the high volume of patients in the emergency department cause the waiting time for test results to be longer than the ideal time [35]. The absence of a blood bank in the hospital also results in complete dependence on external services, thereby prolonging waiting times. Supporting infrastructure such as an internal blood bank is urgently needed to expedite processes and enhance preparedness in emergency situations [36].

The second factor in the results of this study highlights the patient and family factors, including cultural beliefs, lack of medical knowledge, and limited capacity for quick decision-making, which prolong the process of obtaining informed consent from the family. In this context, belief in myths remains a significant challenge in handling emergency caesarean delivery cases, especially in patients with clear medical indications for the procedure. The socio-cultural atmosphere that has developed in society, especially among pregnant women, includes negative perspectives and stigma towards cesarean deliveries [37]. The stigma is that a mother who gives birth via caesarean section is not considered a complete mother if she has not experienced a normal delivery, her breast milk is difficult to produce, and her baby is more susceptible to illness [38, 39]. The stigma related to cesarean delivery can negatively affect the experiences shaped by patients due to the lack of information provision [40].

Furthermore, from the interview results, it was found that the psychological aspects of the patient also affect the smoothness of the caesarean section procedure in emergency situations. The informant stated that patients often feel shocked and experience stress when informed that they must undergo surgery on short notice. This emotional reaction can slow down the decision-making process, especially in giving consent for medical procedures [41]. The delay in the husband’s presence also contributes to the delay in signing the informed consent, which can add more than 30 min, a crucial time in emergency situations [42]. Furthermore, education level plays an important role in health literacy and can influence patient involvement in the medical decision-making process, including in emergency situations such as a caesarean section. This is consistent with previous research conducted by Abdulbaki et al. (2024) [43]. The financial aspect also emerges as one of the significant barriers to making quick decisions for emergency caesarean actions, as conveyed by the informants, many patients still do not have insurance or are worried about the heavy financial burden. Other studies also state that the lack of health insurance and financial concerns in accessing services, even among those who have insurance, are related to delays in seeking emergency care [44]. For non-medical factors that also contribute to delays in the childbirth process, the unpreparedness of the patient’s delivery supplies is a significant issue. Informants reported that cases are still frequently found where patients arrive at the hospital without bringing essential delivery supplies, such as change of clothes, baby diapers, or administrative documents [45].

The third factor in this research highlights the staff factor, among which informants conveyed that the delay in the arrival of the anaesthesiologist or the doctor responsible for the patient can hinder the preparation and execution of the surgery. This finding is in line with other research that states that the performance of caesarean sections in emergency conditions often does not meet the recommended time standards, with a quarter of emergency caesarean sections successfully performed within 75 min. This is due to the type of medical personnel performing the surgery, which has been shown to be related to the length of the interval between decision-making and the execution of the procedure [46]. In line with that, the tiered communication system in teaching hospitals can also be one of the factors causing delays in clinical decision-making. Informants explained that the case reporting process in teaching hospitals must follow a strict hierarchical structure, starting from junior residents, then to mid-level residents, followed by chief residents, and finally to the attending physician [47]. The next obstacle is suboptimal communication and teamwork, especially during shift changes, which becomes one of the factors causing delays in emergency medical response actions, resulting in important clinical information not being conveyed properly. In fact, the clinical handover between shifts in maternity services is a crucial communication moment, with a significant impact on the overall safety of maternity services globally [48]. This obstacle becomes even more complex when considering the limited skills of healthcare workers, particularly midwives who have never undergone maternal and neonatal emergency training. Informants reported that the lack of training affects the medical staff’s ability to recognize critical situations and make quick and accurate decisions. Other research also highlights the importance of healthcare providers specifically trained in emergency obstetric and newborn care, especially in countries with high maternal mortality rates [49].

The fourth factor, which is also the last factor that hinders achieving the response time for emergency caesarean, originates from the internal factors of the hospital. From the document review by the researcher regarding the emergency caesarean policies of five hospitals, only one hospital has those standard operating procedures. This is in line with the response of one of the informants who stated that in the review of emergency Standard Operating Procedures in four districts/cities she visited, most of the Standard Operating Procedures were not developed based on actual needs and clinical practices but rather copied or compiled for administrative purposes such as accreditation. As a result, the medical staff who are supposed to implement the Standard Operating Procedures actually experience confusion due to a lack of understanding of the content and relevance of the procedures.

The impact that will occur if there is no available measurement indicator for emergency caesarean services is that complaint handling cannot be properly addressed, resulting in a negative patient experience that will affect both the physical and psychological well-being of mothers during childbirth [50], maternal and infant mortality that will decrease patient trust and the hospital’s image, as well as a decline in hospital revenue [51]. The other studies found that the recommended 30-minute guideline for caesarean surgery is not met, leading to poor postoperative outcomes [52].

Importantly, this study provided quality improvement strategies conveyed by participants, such as emotional support for patients and families, which were identified as important elements for providing a comforting environment, building trust in the medical team, and reducing the risk of postpartum blues. Other studies highlight that emotional support is one of the strongest determining factors in shaping a good or bad experience during a patient’s hospital stay [53, 54].Clear education and effective communication are also a priority to ensure that patients and families understand the urgency and medical consequences, so that decisions can be made quickly [55]. Furthermore, the policy to prioritise handling the most urgent cases, such as treatment decisions, will be adjusted based on the severity level, with the most critical cases receiving treatment first. In line with the research by Moudi et al. (2020), the findings indicate that obstetric triage is a process involving clinical decision-making to prioritise pregnant women and their foetuses based on the severity and urgency of their conditions in order to allocate medical resources and care appropriately. The results of this study can be used as a basis for the design and implementation of an obstetric triage system [56]. Another small but highly influential factor in achieving emergency caesarean response time is the container that holds essential supplies such as clothing, medications, and necessary medical equipment. In a category one caesarean situation, the container can be immediately used to ensure a quick and efficient response. In line with the research conducted at Riley Mother and Baby Hospital Wing, Eldoret, Kenya, the implementation of this E-kit successfully reduced the maternal mortality ratio by approximately 30% [57]. These overall efforts are designed to create a more responsive and efficient system, which not only prioritizes the safety of mothers and infants but also enhances the patient experience during emergency procedures.

Limited studies in this research indicate that qualitative research results are difficult to generalize to a broader population because participants are only limited to those who agree to be interviewed, which can introduce selection bias. Additionally, this research lacks data sources such as the unavailability of Standard Operating Procedure documents regarding emergency caesarean, so the researcher could not delve deeper into the normative factors contributing to the delay in response time for emergency caesarean sections. Future studies can conduct research using quantitative methods such as regression analysis on the relationship between adherence to Standard Operating Procedures and the effectiveness of achieving emergency caesarean response time.

In conclusion, the results of this study identified four main factors, namely: system factors, patient and family factors, staff factors, and internal hospital policy factors. These four factors are interconnected and contribute to the success or delay in achieving a response time of ≤ 30 min, which ultimately can directly impact the quality of the patient experience. The unavailability of clear Standard Operating Procedures, inadequate facilities, staff delays, ineffective communication, and the lack of understanding among patients and families were the main obstacles identified in this study. To address these issues, informants suggested quality improvement strategies such as the importance of patient education, therapeutic communication, emotional support, and the optimisation of hospital systems and facilities. Thus, achieving the emergency caesarean response time does not only depend on technical aspects but is also greatly influenced by the readiness of human resources and interprofessional coordination systems. In line with that, the commitment of hospital management and the formulation of policies based on real practices are also very much needed to create a positive patient experience and prevent adverse impacts on the safety of mothers and babies. This research is also expected to make a significant contribution to the Indonesian Ministry of Health in optimizing the response time for emergency cesarean operations, thereby delivering better health service through patient-centered care.

## Supplementary Information

Supplementary Material 1.

Supplementary Material 2.

## Data Availability

The data that support the findings of this study are available from Doctoral Program Public Health Faculty of Hasanuddin University upon reasonable request. Requests for access to these data should be directed to corresponding author.
