# Supplementary material for: Exploring health workers’ perspectives on factors affecting patient experience in emergency caesarean section response time: a qualitative study in hospitals in Makassar City, Indonesia
Source: BMC Health Serv Res. 2025 Oct 3;25:1293. doi: 10.1186/s12913-025-13294-4 (PMC12492883; doi:10.1186/s12913-025-13294-4)
Supplement: Supplementary file 1 — Supplementary Material 1. [file 12913_2025_13294_MOESM1_ESM.docx]

**INTERVIEW GUIDELINE**

Assalamu Alaikum Wr. Wb / Shalom / Good morning / afternoon, Madam.

Thank you for your willingness to participate in the research titled **"Factors Affecting Patient Experience in Emergency Caesarean Response Time."** Currently, the national hospital accountability report on emergency caesarean response time is far below the target. Out of the planned target of 331 hospitals, only 58 (17.5%) have been able to meet the required response time. This issue can significantly impact patient experience, including psychological factors and adverse effects on the health of both mothers and infants. Therefore, the aims of this study is to explore the factors that affecting patient experience related to emergency caesarean response time.

This research will go through several stages, namely: 1) Exploring the factors contributing to delays in emergency caesarean response time that affect patient experience. 2) Identifying strategies to address these issues.

Stages 1

1. During your profession as an obstetrician-gynecologist, general practitioner, nurse, or midwife in this hospital, have you encountered any obstacles in achieving the emergency caesarean response time of ≤ 30 minutes? According to Sonis (2019), there are several factors that may act as barriers, namely:

a. Systems Factors
b. Patient Factors
c. Staff Factors
d. Leadership & Policy Factors

Stage 2

1. How do you address or minimize these obstacles? Additionally, what are the best practices that healthcare professionals should implement to ensure patients have a positive experience during the emergency caesarean response time?
