# Supplementary material for: Exploring health workers’ perspectives on factors affecting patient experience in emergency caesarean section response time: a qualitative study in hospitals in Makassar City, Indonesia
Source: BMC Health Serv Res. 2025 Oct 3;25:1293. doi: 10.1186/s12913-025-13294-4 (PMC12492883; doi:10.1186/s12913-025-13294-4)
Supplement: Supplementary file 2 — Supplementary Material 2. [file 12913_2025_13294_MOESM2_ESM.docx]

| Characteristics | Total | |
| --- | --- | --- |
|  | N | % |
| Total | 14 | 100.0 |
| **Sex** |  |  |
| Men | - |  |
| Women | 14 | 100 |
| **Age (years old)** |  |  |
| 30-39 | 7 | 50.0 |
| 40-49 | 4 | 28.5 |
| ≥50 | 3 | 21.5 |
| **Occupation** |  |  |
| Obstetrician and gynecologist | 4 | 28.5 |
| General Practioner | 2 | 14.3 |
| Midwifery | 6 | 42.9 |
| Nurse | 2 | 14.3 |
| **Type Hospital** |  |  |
| General Hospital | 6 | 42,9 |
| Mother and Child Hospital | 8 | 57,1 |
| **Period of work experience** |  |  |
| 2-10 yr | 7 | 50.0 |
| 11-20 yr | 6 | 42.9 |
| ≥21 yr | 1 | 7.1 |

Supplementary table 2. Characteristics of study participants by profession
